# Effects of continuous midwifery labour support for women with severe fear of childbirth

**DOI:** 10.1186/s12884-015-0548-6

**Published:** 2015-05-15

**Authors:** Gunilla Sydsjö, Marie Blomberg, Sofie Palmquist, Louise Angerbjörn, Marie Bladh, Ann Josefsson

**Affiliations:** Department of Obstetrics and Gynaecology and Department of Clinical and Experimental Medicine, Faculty of Health Sciences, Linköping University, Linköping, Sweden; Department of Obstetrics and Gynaecology, University Hospital, SE-581 85 Linköping, Sweden

**Keywords:** Fear of childbirth, Support, Obstetric outcome

## Abstract

**Background:**

Continuous support by a midwife during childbirth has shown positive effects on the duration of active labour, use of pain relief and frequency of caesarean section (CS) in women without fear of childbirth (FOC). We have evaluated how continuous support by a specially assigned midwife during childbirth affects birth outcome and the subjective experience of women with severe FOC.

**Methods:**

A case–control pilot study with an index group of 14 women with severe FOC and a reference group of 28 women without FOC giving birth. In this study the index group received continuous support during childbirth.

**Results:**

The women with severe FOC more often had an induction of labour. The parous women with severe FOC had a shorter duration of active labour compared to the parous reference women (*p* = 0.047). There was no difference in caesarean section frequency between the two groups. Women with severe FOC experienced a very high anxiety level during childbirth (OR = 20.000, 95 % CI: 3.036-131.731).

**Conclusion:**

Women with severe FOC might benefit from continuous support by a midwife during childbirth. Midwives should acknowledge the importance of continuous support in order to enhance the experience of childbirth in women with severe FOC.

## Background

During pregnancy a certain amount of anxiety is considered a normal part of the preparation for the upcoming birth [1]. In some women this feeling develops into fear of childbirth (FOC) which might have many negative consequences [2]. These include prolonged duration of active labour, greater use of pain relief, higher rate of emergency Caesarean sections, more negative personal experiences, and even the wish that caesarean section (CS) had been available as an elective option [3–7]. In Sweden severe FOC affects 3–16 % of women [4, 8]. Women are screened for FOC at the antenatal healthcare clinic and those with FOC are referred to a psychosocial unit at the Department of Obstetrics and Gynaecology. Where they receive extended support including as psycho education and cognitive behavioural therapy from a team of midwives, obstetricians and psychologists.

In women without FOC, previous studies have shown that continuous support by a midwife or nurse during labour has positive effects on the duration of active labour, use of pain relief and frequency of CS [9, 10]. Also, these women experience more control during childbirth, have fewer negative feelings about the birth and will often prefer to have such support in a future labour [11]. The aim of this pilot study was to evaluate how continuous support by a specially assigned midwife during labour affects birth outcome and subjective experience in women with severe FOC.

## Methods

The index group consisted of 14 women with severe FOC and who gave birth at a Swedish university hospital. The women communicated their fear at the antenatal healthcare clinic and were referred to an obstetrician at the psychosocial unit of the Department of Obstetrics and Gynaecology. The women were diagnosed with severe FOC according to DSM-IV, i.e., the American Psychiatric Associations diagnostic criteria for severe phobia [12]. They all received the standard counselling based on psycho-education and cognitive behaviour therapy during pregnancy offered to women with FOC. In addition, they were offered continuous support by one of two specially assigned midwives during labour, if they were planned for a vaginal birth. Continuous support was defined as the presence of an experienced midwife with specific training in psychological treatment of women with FOC throughout the labour and birth. The women with FOC met with the selected midwife during the last trimester to establish a trustful relation and to provide the woman with an opportunity to become familiar with the delivery ward. The continuity of care was implemented according to the following protocol:

1. The woman was invited to the delivery ward for a first meeting with the selected midwife. The partner was also welcomed.
2. At the first meeting the delivery ward was presented and the delivery rooms were shown. All equipment was explained and tested if this was asked for. In order to meet specific fears or questions and to prepare the woman for events that might occur during the childbirth an external and sometimes a vaginal examination were offered as well as a CTG (cardiotocogram). The examinations and the foetal surveillance were offered in order to expose the women to these routines and thus help the women to get used to exposures that might be fearful to them. Questions were encouraged and the birth process was thoroughly discussed.
3. A second visit with the selected midwife at the delivery ward was offered if the woman so wished.
4. The woman was encouraged to contact the midwife by telephone or schedule additional visits to the delivery ward for further support or information if needed.
5. If a birth plan /contract had not been established together with the obstetrician in routine prenatal care of FOC the midwife and the woman wrote an appropriate document.
6. Information was given to each woman that the two assigned midwives would do their utmost to attend and assist the woman’s childbirth but that circumstances such as illnesses and other unaccounted situations may occur and in these circumstances a regular midwife would assist the woman according to the birth plan.
7. Individual support was continuously offered to the two assigned midwives by the obstetrician in charge at the psychosocial unit.

As a reference group, 28 women without FOC according to antenatal care records who gave birth at the same hospital and day as the index women and who received standard labour and birth care were retrospectively chosen.

In order to evaluate the obstetric outcomes, data were extracted from the women’s antenatal and birth records. To explore the woman’s subjective childbirth experience, a structured telephone interview was conducted. After six months, a letter containing information about the telephone interview and emphasizing that participation was voluntary was sent to women both in the index and in the reference group. The interviews were structured and were performed by two medical students in their final year. We chose this approach in order to minimize bias. The students did not have any connection with the treatment of the women and was not involved in implementation of the study. They were specially trained by the psychotherapist in the research team to conduct the interviews. Each woman was interviewed over the telephone. Two women in the index group were excluded, as they did not receive an assigned midwife at the delivery ward due to unknown reasons. One index woman refused to participate in the telephone interview, as she was very unsatisfied with the treatment provided. Two of the women in the control group also declined participation and expressed a negative birth experience as the reason. Finally 11 index women and 26 control women were interviewed. A structured interview guide was developed by the research team and used in the interviews, in order to cover all areas of interest.

The background maternal characteristics derived from the women’s records were maternal age, body mass index (BMI), civil status, occupation, smoking, parity (present pregnancy not included), previous miscarriage, and previous legal abortion.

The obstetric characteristics derived from the delivery records of the present pregnancy were gestational week at childbirth, induction of labour, oxytocin augmentation of labour and Apgar score at 5 min.

The outcome measures of the study were duration of active labour in primiparous and parous women, use of pain relief and frequency of emergency CS and the woman’s subjective experience of the childbirth. Onset of active labour was defined as regular painful uterine contractions, three to four in a 10 min period, and a cervical dilation of three centimetres or more, according to Swedish standard, practiced at all maternity units. The midwife at the delivery ward recorded the time when the regular contractions started. If regular contractions had started at home, the pregnant woman reported the time. Duration of labour was the time between onset of labour and the time of birth. Epidural anaesthesia, paracervical local anaesthesia, nitrous oxide, acupuncture, intravenous morphine were classified as pain relief.

All statistical analyzes were performed using IBM SPSS Version 19 (Armonk, NY, USA). Statistical analyzes included Pearson’s chi-square, Fisher’s exact test and Student’s *t*-test. Logistic regressions were made with each outcome measure as dependent variable. The answering alternatives of the interview questions were re-categorized into two alternatives when necessary. In all analyzes, a two-sided *p*-value of ≤ 0.05 was considered significant.

The women gave oral informed consent before participating in the study. The study outline was approved by The Regional Ethical Review Board in Linköping.

## Results

The two groups were similar in terms of maternal age, BMI, civil status, occupation, smoking, parity (present pregnancy not included), earlier spontaneous abortion, and earlier legal abortion. There were no differences in obstetric characteristics such as gestational week at childbirth, augmentation of labour and Apgar score at 5 min (Table 1). However, more women with severe FOC experienced an induction of labour.

**Table 1:** Characteristics of the study population

	Index group		Reference group		*p*-value
	n	%	n	%	
Maternal age					
Mean/SD	31.6/5.7		29.2/3.4		0.214
BMI					
Mean/SD	27.7/5.2		24.5/3.5		0.086
Civil status					
Married/Cohabiting	10	90.9	26	100.0	0.297
Single	1	9.1	0	0.0	
Permanently employed					
Yes	8	72.7	22	84.6	0.403
No	3	27.3	4	15.4	
Smoking					
Yes	0	0.0	0	0.0	-
No	11	100.0	26	100.0	
Parity (present pregnancy not included)					
0	3	27.3	8	30.8	1.000
1	8	72.7	18	69.2	
Miscarriage					
Yes	2	18.2	5	19.2	1.000
No	9	81.8	21	80.8	
Legal abortion					
Yes	3	27.3	2	8.0	0.154
No	8	72.7	23	92.0	
Gestation at childbirth					
<37 weeks	1	9.1	1	3.8	0.512
≥37 weeks	10	90.9	25	96.2	
Induction of labour					
Yes	7	63.6	3	11.5	0.003
No	4	36.4	23	88.5	
Oxytocin augmentation in labour					
Yes	9	81.8	14	53.8	0.150
No	2	18.2	12	46.2	
Apgar score at 5 min					
<7	0	0.0	0	0.0	-
>7	11	100.0	26	100.0	

Table 2 shows the result of the bivariate analyzes of all outcome measures. No differences were found concerning duration of active labour in primiparous women, the use of pain relief or the frequency of emergency CS. Concerning duration of active labour in parous women, a difference was found with longer duration in the reference group. Regarding the subjective delivery experience, a significant difference was found concerning the question “*What level of anxiety did you suffer from during your childbirth?”* Logistic regression analyzes showed that women in the index group reported a higher level of anxiety (OR = 20.000, 95 % CI: 3.036-131.731). In these analyses, no other factors were associated with the level of anxiety. No differences were found between the two groups concerning the other questions of the subjective childbirth experience.

**Table 2:** Childbirth outcome

	Index group n = 11		Reference group n = 26		*p*-value
	n	%	n	%	
Duration of active labour in minutes (CS excluded)					
Mean/SD primiparous women	565.0/-^a^		510.0/350.1		0.888
Mean/SD parous women	233.0/123.1		366.9/157.0		0.047
Pain relief					
None	0	0.0	3	11.5	0.540
Epidural anaesthesia	8	72.7	13	50.0	0.285
Nitrous oxide	7	63.6	22	84.6	0.203
Other	4	36.4	6	23.1	0.442
Emergency caesarean section					
Yes	3	27.3	1	3.8	0.070
No	8	72.7	25	96.2	
How did you experience your childbirth?					
Positive	9	90.0	20	87.0	1.000
Negative	1	10.0	3	13.0	
How did your childbirth turn out in relation to your prior expectations?					
Better than expected	7	63.6	9	34.6	0.209
As expected	1	9.1	8	30.8	
Worse than expected	3	27.3	9	34.6	
What level of anxiety did you suffer from during your childbirth?					
Low	2	20.0	21	80.8	0.001
High	8	80.0	5	19.2	
How did you consider the effect of pain relief during childbirth?					
Good	8	88.9	17	81.0	1.000
Poor	1	11.1	4	19.0	
Index women: Would you recommend a friend to have a specially assigned midwife during childbirth?					
Yes	11	100.0	-	-	-
No	0	0.0	-	-	

^a^Data of one index woman only

## Discussion

To our knowledge, this is the first study to investigate the effect of continuous support by a specially assigned midwife in women with severe FOC.

Previous studies have shown negative effects of severe FOC on duration of active labour, use of pain relief and frequency of CS [3–5]. It has also been shown that continuous support during labour and birth has positive effects on the factors mentioned above in women without FOC [9, 10]. The fact that the present study showed similar results in the two groups concerning use of pain relief and frequency of CS indicates that women with severe FOC may benefit from continuous support by a midwife during labour. The overall frequency of epidural use during the study period was 36 % at the delivery ward, which is lower than both the index and the control group. The overall use of nitrous oxide at our department was 87 % which is in accordance with the use in the control group but higher than in the index group. An explanation could be that nitrous oxide is avoided among women with FOC due to their earlier experiences of lack of control and blackouts during previous deliveries. The higher prevalence of epidural in the index group than the overall prevalence is not surprising since fear of pain is more frequent in the group of women with FOC and effective pain relief is the premise for a successful vaginal delivery.

The shorter duration of active labour seen in the parous women with severe FOC further strengthens this conclusion. Only one primipara in the index group was delivered vaginally and therefore no comparisons are available for this variable. An explanation to the shorter duration of active labour was that the women with severe FOC might have been given trustful support and a more active management of delivery. This assumption is strengthened by the fact that the women with severe FOC in spite of the higher frequency of inductions of labour still had a shorter active phase of labour.

In a recent review by Sandall *et al.* 2013 it was found that midwife-led models for care to pregnant women show a overall positive effect on women’s health and obstetric and neonatal outcomes [13].

There is evidence that women with severe FOC experience their childbirth more negatively than women in general [6, 7]. It has also been shown that continuous support during delivery has positive effects on the subjective delivery experience in women *without* FOC [11]. The present results showed no differences between the two groups concerning the questions *“How did you experience your childbirth?”, “How did your childbirth turn out in relation to your prior expectations?”* and “*How did you consider the effect of pain relief during childbirth?”.*

This indicates positive effects of the continuous support, which is strengthened by the fact that all the women with severe FOC stated that they would recommend a friend to have a specially assigned midwife during childbirth.

Around 30 % of the women giving birth in both groups had a worse childbirth experience than they expected. This result could be looked upon from two different views. One woman out of three with FOC experienced a worse childbirth than expected which was equally to the reference groups’ experience. The expectancy could be that a larger proportion of women with FOC should have had worse experience than expected without the continuous midwife support. On the other hand the amount of women in the reference group with a worse experience than expected was surprisingly high. This result needs to be evaluated further since we do not want women to develop a secondary FOC or to worsen an already present but not reported fear. A secondary FOC also poses an increased risk for a prolonged time to subsequent pregnancy [14].

A strength of this small pilot study is that the outcome measures include both objective and subjective aspects of the effects of continuous support on childbirth outcome. The fact that the two groups were similar concerning all background factors except induction of labour further strengthens the study. Potential limitations were the facts that the study was non-randomized with a small sample, but on the other hand it was designed as a pilot study. Another limitation was that the women with severe FOC were compared to a reference group of women without FOC. If two groups of women with FOC had been compared, the effects of the continuous midwifery support might have appeared more clearly. Also, no variable on the specific amount of time the midwives spent supporting the delivering women was available. Since this was a pilot study, further research on this subject is needed.

## Conclusion

Women with severe FOC might benefit from continuous support by a midwife during childbirth. Midwives should acknowledge the importance of continuous support in order to enhance the experience of childbirth in women with severe FOC. A prospective randomized trial with a larger sample and women with FOC in both index and reference group would be of great value.
